# Design of a Differential Low-Noise Amplifier Using the JFET IF3602 to Improve TEM Receiver

**DOI:** 10.3390/mi13122211

**Published:** 2022-12-13

**Authors:** Shengjie Wang, Yuqi Zhao, Yishu Sun, Weicheng Wang, Jian Chen, Yang Zhang

**Affiliations:** 1 Key Laboratory for Geophysical Instrument of Ministry of Education, Jilin University, Changchun 130061, China; 2 College of instrumentation & Electrical Engineering, Jilin University, Changchun 130061, China; 3 Engineering Research Center of Geothermal Resources Development Technology and Equipment, Ministry of Education, Jilin University, Changchun 130026, China

**Keywords:** low noise, junction field-effect transistor, transient electromagnetic method

## Abstract

The observed data of transient electromagnetic (TEM) systems is often contaminated by various noises. Even after stacking averages or applying various denoising algorithms, the interference of the system noise floor cannot be eliminated fundamentally, which limits the survey capability and detection efficiency of TEM. To improve the noise performance of the TEM receiver, we have designed a low-noise amplifier using the current source long-tail differential structure and JFET IF3602 through analyzing the power spectrum characteristics of the TEM forward response. By the designed circuit structure, the JFET operating point is easy to set up. The adverse effect on the JFET differential structure by JFET performance differences is also weakened. After establishing the noise model and optimizing the parameters, the designed low-noise differential amplifier has a noise level of $0.60\mathrm{nV}/\sqrt{\mathrm{Hz}}$, which increases the number of effective data 2.6 times compared with the LT1028 amplifier.

## 1. Introduction

The transient electromagnetic (TEM) method is a classic geophysical exploration method that detects the induction response of a geological body to emitted electromagnetic (EM) waves. It distinguishes subsurface geological structures based on characteristic differences in the amplitude and decay rate of the response of geological bodies with different resistivities. Due to the non-destructive propagation of EM waves in the subsurface medium, TEM is widely used in mineral resource exploration, geological surveys, and urban disease exploration. The TEM signal is characterized by high amplitude in the early time and low amplitude in the late time. Therefore, in order to obtain a high-quality signal, it is necessary to use a wide frequency band, a large dynamic range amplifier, and a high-precision acquisition system with high speed to collect high-quality TEM data for subsequent data processing and data interpretation [1,2].

The observed signal is always interfered with by strong noise in practice. The noise contaminates the signal, especially the late time, which is in low amplitude and represents the deep geological information [3]. These kinds of noise are classified by their source. Part of the noise is from the EM interference in the environment, which is called environmental noise; the other part of the noises is from the TEM receiver, which is called the system noise floor. Environmental noise has a certain pattern in statistics and can be eliminated by using algorithms such as half-cone gate filtering, minimum noise separation, and improved algorithms based on temporal correlation [4,5,6]. However, these data processing methods cannot eliminate the effect of the system noise floor, so the TEM receiver needs to be optimized to obtain a more accurate signal.

The amplifier noise floor of the TEM receiver is a major part of the system noise floor, and there have been many related studies on TEM low-noise amplifiers (LNA) [7]. Chen et al. designed an amplifier with a noise floor of $1.83\mathrm{nV}/\sqrt{\mathrm{Hz}}$ using the low-noise integrated operational amplifier (IOA) AD797 for the ZTEM receiving device [8]. Pi et al. used the IOA LT1028 to design a low-noise preamplifier circuit with an ideal noise of $2.45\mathrm{nV}/\sqrt{\mathrm{Hz}}$ for urban TEM devices [9]. Low-noise IOA is mainly used in TEM preamplifiers currently [10,11], but the noise characteristics of IOAs are inferior to discrete components due to the cost restriction, which also limits the noise optimization of subsequent circuits [12]. To solve the problem, Wang et al. developed a $2\mathrm{nV}/\sqrt{\mathrm{Hz}}$ ZTEM signal conditioning circuit using the junction field-effect transistor (JFET) LSK389B in the helicopter TEM receiver, which introduced a new idea for the optimization of TEM devices [13].

At present, JFET LNA is mainly used for the measurement and amplification of weak signal sensors such as piezoelectric accelerometers, wireless radio frequency equipment, and seismic accelerometers at present [14]. Scandurra proposed a feedback compensation JFET differential amplifier circuit for low-frequency noise measurement circuits with the noise floor of $1.00\mathrm{nV}/\sqrt{\mathrm{Hz}}@1.00\mathrm{kHz}$ [15]. Cannatà designed a $0.80\mathrm{nV}/\sqrt{\mathrm{Hz}}@1.00\mathrm{kHz}$ single-ended amplifier based on discrete components JFET for low-frequency noise measurement [16]. To summarize, JFET has a very low system noise floor and a wide stable noise frequency band [17], which is very suitable for noise optimization of TEM receivers.

The rest of the paper is as follows: In Section 2, we analyze the time-frequency characteristics of the TEM signal according to the TEM principle and forward response, and clarify the requirements; Section 3 gives the JFET LNA for TEM receiver with the circuit structure, model, and actual noise floor test; In Section 4, we conducted laboratory experiments to compare the length of TEM effective data from the JFET receiver and the IOA receiver. Finally, the optimization direction of the amplifier and the application effect in transient electromagnetic systems are discussed.

## 2. The Analysis of TEM Signal

**Figure 1:**
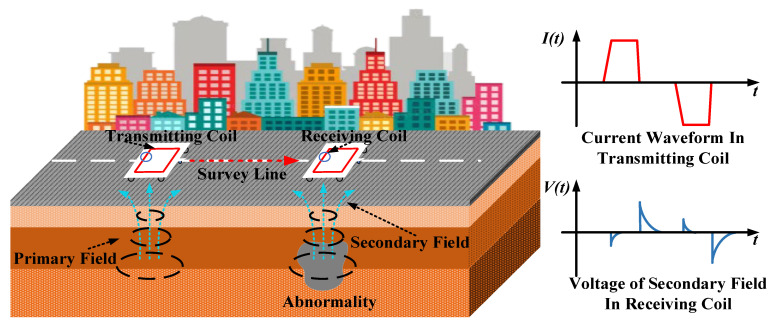
Schematic diagram of TEM survey.

The TEM system consists of a transmitting coil, a transmitter, a receiving coil, and a receiver. Its working principle is shown in Figure 1. A bipolar step pulse current is sent through the transmitting coil by the transmitter. Meanwhile, a primary field is created, surrounded, and transmitted in the form of smoke rings. Due to Faraday’s law of electromagnetic induction, geological bodies are excited by the primary field, and the eddy current is induced underground with the secondary field when the pulse is on. When the pulse is turned off, the secondary field starts decaying at different rates, which are related to the conductivity of the subsurface layers. The voltage in the receiving coil is generated by the secondary field and observed in the receiver. After TEM data interpretation, the abnormality and subsurface layers can be known [18].

**Figure 2:**
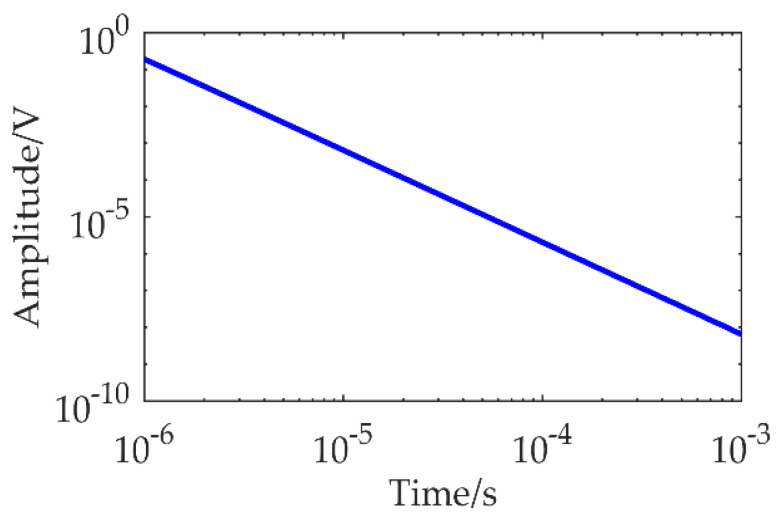
The TEM forward response.

**Figure 3:**
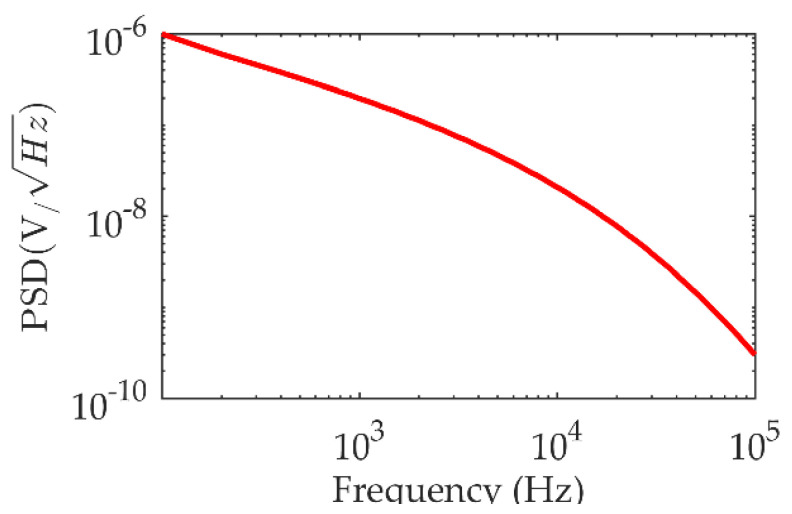
The PSD of TEM forward response.

To further analyze the characteristics of TEM signals, we use the TEM forward modeling to generate ideal signals for time-frequency analysis. Since the actual geological layering is very complex, it is difficult to fully cover the condition of multi-layer forward modeling. Because the TEM has the characteristics of a strong response in a low-resistance medium and a weak response in a high-resistance medium, the actual geological response will be higher than the pure high-resistance response and lower than the pure low-resistance response. Therefore, we set the high-resistance uniform half-space to 100 Ω·m and adjust the transmitting magnetic moment to 1 × 10^4^ A·m^2^, and the receiving equivalent area to 128 m^2^ [19]. The forward modeling based on the sinusoidal numerical filtering algorithm is used to obtain the forward response within 1 ms, as shown in Figure 2 [20,21]. It is observed that the early TEM forward response is five orders of magnitude higher than the late response. Therefore, to identify the late signal, not only a high-amplification amplifier is required, but also the noise floor of the amplifier is limited; otherwise, it is extremely difficult to obtain useful information on TEM from the circuit noise. Since the noise power spectrum is mainly used to measure the noise characteristics of LNA, we calculate the power spectral density (PSD) of the forward response in 100 Ω·m pure high resistance as a reference index for designing the LNA. The results in Figure 3 show that the low-frequency PSD of the TEM signal is larger and the high-frequency PSD is smaller and below $1\mathrm{nV}/\sqrt{\mathrm{Hz}}$. Therefore, when designing the amplifier, it is necessary to ensure that the amplifier has a stable gain and an extremely low noise floor in the whole frequency band to obtain high-quality TEM data and lay the foundation for subsequent data denoising and data interpretation.

## 3. Optimal Design of JFET LNA

### 3.1. Selection of LNA Circuit Components

Because of the characteristics of the wideband and large dynamic range of the TEM signal, the suitable amplifier must have a sufficient gain-bandwidth product, as mentioned, and a low-noise cascade amplifier circuit needs to be designed to achieve reliable signal amplification. The Frith formula, shown in Equation (1), reflects the relationship between the overall noise of the cascaded circuit and the noise of the circuits at all levels as follows:

(1) $$F=F_{1}+\frac{F_{2}-1}{K_{1}}+\frac{F_{3}-1}{K_{1}K_{2}}+\cdots +\frac{F_{M}-1}{K_{1}K_{2}\cdots K_{M-1}}\begin{array}{cc} & \left(M=2,3,\cdots \right) \end{array}$$

*F* is the total noise figure of the cascaded amplifier, *F_M_* is the noise figure of the *M* amplifier, and *K_M−_*_1_ is the gain of the *M*−1 amplifier. The noise of the cascaded amplifier is mainly restricted by the first-stage amplifier, and the influence of the latter stage is slighter, which means reducing the noise floor of the first-stage amplifier is the primary consideration when designing a low-noise system for weak signal detection.

To optimize the noise floor of the first-stage amplifier circuit, a low-noise device must be selected as the core of the circuit. Common choices of low-noise devices are bipolar transistors (BJTs), JFETs, and IOAs. Because BJT devices and BJT-type IOAs have a low noise floor in low-frequency and mid-frequency bands, they are generally used to make TEM preamplifiers, but with the development of low-noise JFET, such as LSK389B and 2SK3320. These components significantly reduce device voltage noise when their own current noise is negligible. These give low-noise JFETs the advantage of a low device noise floor and simple noise components, which are more suitable for making LNAs. In order to select the low-noise device for the preamplifier, we compared the noise performance of common low-noise devices, and the results are shown in Table 1.

Table 1 shows that IF3602 has very low voltage noise ($0.5\mathrm{nV}/\sqrt{\mathrm{Hz}}@100\mathrm{Hz}$) and the noise frequency is only 100 Hz. The principle of a JFET is using the electric field effect in the semiconductor to change the barrier width of the gate PN junction by controlling the gate-source voltage V_GS_ and using the pinch-off of the depletion layer to control the internal carriers. No current flows between the gate and the source, so the current noise is negligible. This feature simplifies the noise model of the JFET amplifier circuit. According to the comparison, we proposed a low-noise differential circuit design for the JFET IF3602 and combined the noise model to optimize the external circuit.

### 3.2. Overall Design of Low-Noise JFET Amplifier Circuit

**Figure 4:**
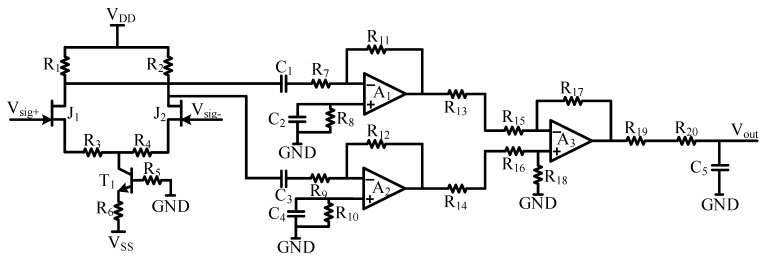
Schematic diagram of low-noise amplifier circuit.

**Figure 5:**
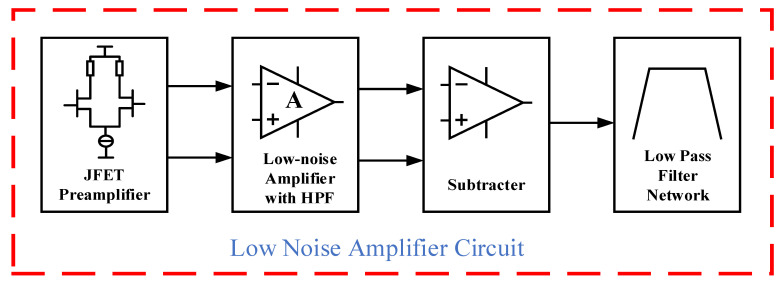
Schematic diagram of the function of the low-noise amplifier circuit.

Based on the characteristics of the TEM signal analyzed in Figure 2 and Figure 3, we designed the amplifier circuit shown in Figure 4. According to the functions of each cascade amplifier, the LNA circuit is divided into a four-level structure, as shown in Figure 5. The first-stage circuit is a differential circuit based on the JFET IF3602, which can achieve low-noise and high-gain amplification for the received signal. The low noise floor of the amplifier circuit means that the impact of circuit noise on the signal-to-noise ratio (SNR) of the TEM signal is weak. Besides, the high gain in the first-stage circuit weakens the impact of the noise floor in the post-stage circuits. For the differential circuit, it also has the characteristics of high differential mode magnification and low common mode magnification, which can effectively suppress the interference of common mode noise and enhance the anti-interference ability of the TEM device.

The second-stage circuit is an amplifier made from the low-noise IOA LT1028 with a high-pass filtering function. Although the noise floor of the LT1028 IOA is not as good as IF3602, its equivalent voltage noise is only $0.85\mathrm{nV}/\sqrt{\mathrm{Hz}}$, and the IOA circuit is easy to design because of its simple structure. Therefore, it is very suitable to be the secondary amplifier. In addition, as shown in Figure 3, it is necessary to reduce the impact of a low-frequency component of TEM to avoid the early signal saturation. Therefore, adding high-pass filtering can make full use of its amplitude-frequency characteristics, to reduce the amplification factor of low-frequency components, and avoid early signal saturation.

The third-stage circuit contains a precision differential amplifier INA105, which integrates a laser-corrected 25 kΩ precision resistor to ensure the gain accuracy of the internal feedback amplifier circuit and reduce the impact of resistance temperature drift on the accuracy of the differential amplifier.

An RC low-pass filter network is used in the fourth stage with a 257 kHz cut-off frequency, which includes the ideal signal frequency band in forwarding modeling, to avoid the influence of high-frequency noise on the circuit and ensure the quality of TEM signals.

### 3.3. Design and Analysis of JFET Differential Low-Noise Amplifier Circuit

As an important part of the LNA, the JFET differential amplifier circuit needs to have the characteristics of low noise and high gain. As a voltage-controlled device, JFET must work in the amplification mode when used for signal amplification. At that time, the depletion layer is partially pinch-off, the input AC signal causes the width of the depletion layer to change slightly, and the output drain current also changes accordingly. The signal is amplified at the output. The noise floor and amplification capability of JFET are also limited by the internal channel. The JFET must be operated at a suitable static operating point to make it both low-noise and high-gain.

In practice, due to the limitations of manufacturing technology and semiconductor doping technology, JFET devices will show a large performance difference even in the same batch [17], resulting in the unequal current in the left and right branches of traditional JFET long-tail differential circuits. This problem makes the circuit design difficult because of the static operating point offset. Fortunately, the IF3602 not only has excellent amplification characteristics and noise performance but also integrates a pair of matched low-noise JFETs, which greatly reduces the impact of JFETs differences on performance. In addition, we introduce a source-coupled differential amplifier circuit design, in which a current source is used to replace the tail resistors in traditional long-tail differential circuits. Compared with the long-tail differential circuit, the higher equivalent resistance of the current source gives the circuit an extremely strong common-mode signal rejection and can provide stable current output for the branch. Usually, the following three correlated variables need to be considered when setting the static operating point of the JFET: drain-source voltage V_DS_, gate-source voltage V_GS_, and drain current I_D_. However, the new source-coupled differential amplifier circuit design reduces the variables to V_DS_ and V_GS_, which simplifies the difficulty of circuit design. The DC path and the AC path were analyzed in this part, where the DC path is used to stabilize the static operating point for the device for optimum performance of the JFET; the AC path determines the amplification capability of the circuit. The results will provide theoretical support for subsequent circuit noise analysis and circuit optimization.

**Figure 6:**
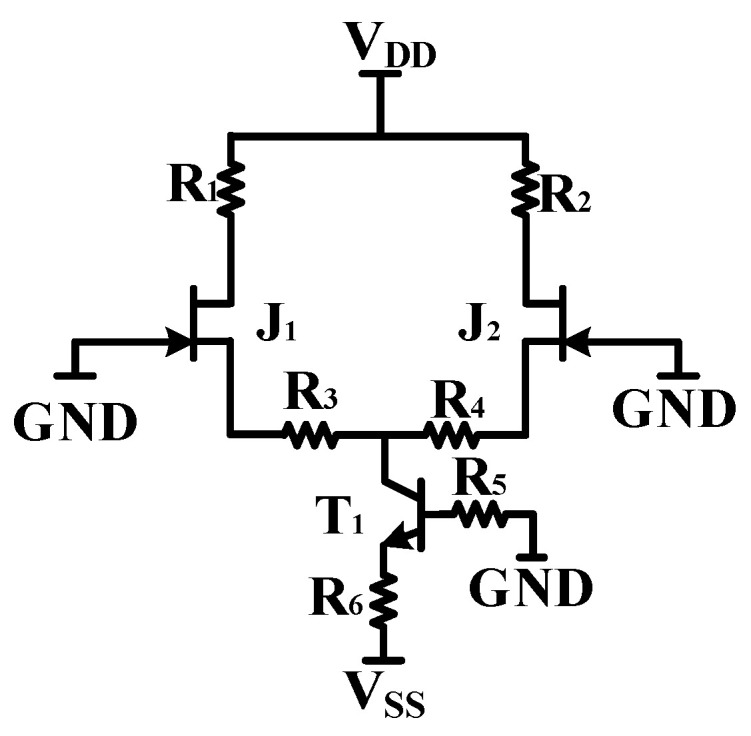
DC equivalent path of JFET differential circuit.

The DC path of the JFET amplifier circuit is shown in Figure 6, where BJT T_1_, R_5_, and R_6_ are formed as a current source to output a stable current of I_p_ = (−V_SS_−V_PN_)/R_6_. Due to the high symmetry of the circuit, I_D1_ = I_D2_ = 0.5I_p_. After determining the quiescent operating current of the JFET, only the operating voltages V_DS_ and V_GS_ need to be considered. For JFET, the stable amplification must be achieved in the amplification mode. It is necessary to keep the V_DS_ > 0, V_GS_ < 0 where V_D_ = V_DD_ − I_D1_R_1_ = V_DD_ − I_D2_R_2_ and I_D1_R_3_ = −V_BE_ + I_D2_R_4_. The JFET gate is grounded during DC analysis, so V_G_ = 0. The current source maintains the stability of the loop current, so I_D1_ and I_D2_ are known. According to the external resistors R_1_, R_2_, R_3_, and R_4_, the static operating point of the amplifier circuit can be calculated, avoiding the problem of static operating point setting caused by the interaction of V_GS_, V_DS_, and I_D_ in the JFET circuit. In order to ensure that the IF3602 has excellent noise characteristics, it needs to be set at the minimum noise static operating point “V_DS_ = 3 V, I_D_ = 5 mA” in the datasheet. After calculating the theoretical external parameters, the R_1_ and R_2_ should be fine-tuned to fit the minimum noise static operating point in the datasheet as much as possible, ensuring that the IF3602 can stably exert its noise performance advantages.

**Figure 7:**
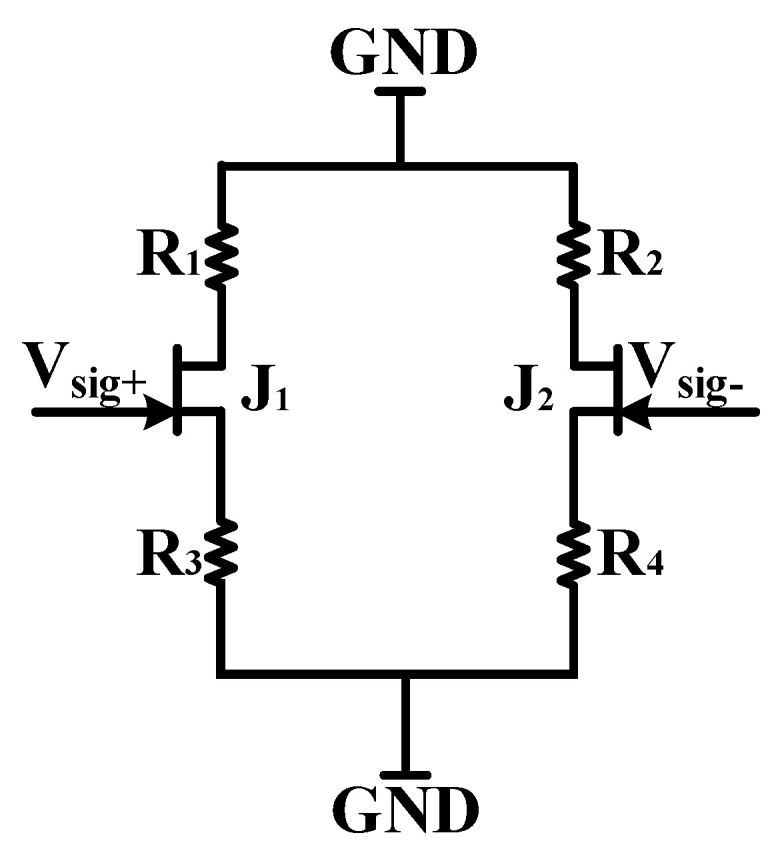
AC equivalent path of JFET differential circuit.

**Figure 8:**
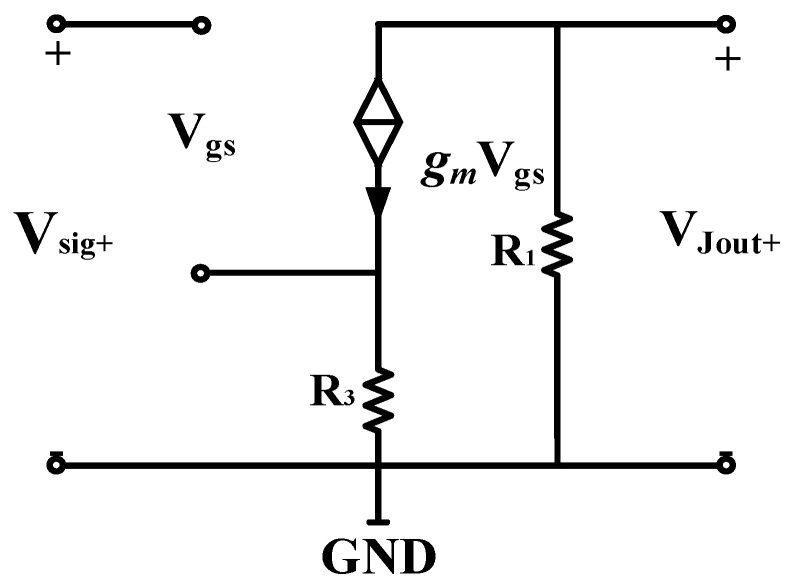
Analysis of AC small signal model of JFET differential circuit.

The AC path of the JFET amplifier circuit is shown in Figure 7. Because the differential circuit contains excellent differential mode signal amplification capability and common mode noise suppression capability, the current source at the common terminal does not be amplified. Therefore, the current source can be ignored in the AC equivalent circuit. Besides, the amplification capabilities of the circuits on both sides are the same, so only one side of the circuit needs to be considered when constructing the small-signal model to analyze the magnification. The single-side small-signal model is shown in Figure 8. It can be seen from Figure 8 that the amplification factor of the differential amplifier circuit satisfies the following:

(2) $$A_{v}=\left(-g_{m}R_{1}\right)/(1+g_{m}R_{3})=\left(-g_{m}R_{2}\right)/\left(1+g_{m}R_{4}\right)$$

*g_m_* is the JFET transconductance, which is affected by the internal conduction channel, so it is only related to the static operating point. The source-coupled differential amplifier circuit constructed by the current source not only simplifies the circuit design but also stabilizes the noise performance and amplification capability of the JFET IF3602. *A_v_* is the magnification of the differential amplifier circuit, and its value mainly depends on the ratio of the external circuits R_1_ and R_3_ or R_2_ and R_4_. The purpose of R_3_ and R_4_ is to stabilize the source potential of the JFET. In order to maintain the amplification ability of the circuit, their values are relatively small (less than 10 Ω). Therefore, maintaining the JFET in the amplification area, keeping the JFET working near the static operating point of minimum noise, and increasing the values of R_1_ and R_2_ at the same time will significantly increase the amplification factor of the differential amplifier circuit and ensure the performance of the first-stage differential amplifier circuit.

### 3.4. Noise Analysis and Parameter Optimization of JFET Differential Amplifier Circuit

**Figure 9:**
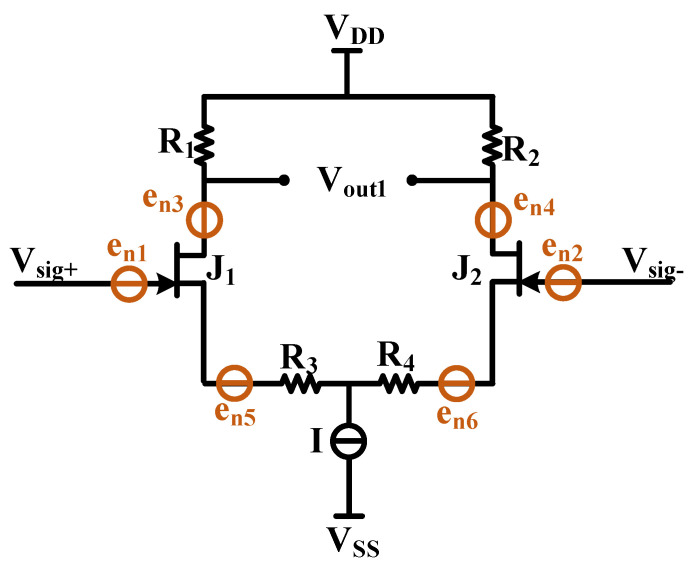
JFET differential circuit noise model.

The equivalent model of the circuit is constructed by analyzing the AC and DC path signals of the circuit. In order to further optimize the noise performance and amplification performance of the JFET IF3602 differential amplifier circuit, a noise equivalent model was built as shown in Figure 9. e_n1_ and e_n2_ are the equivalent noise sources of the IF3602, e_n3_ and e_n4_ are the equivalent thermal noise sources of the drain resistors R_1_ and R_2_, and e_n5_ and e_n6_ are the equivalent thermal noise sources of the source resistors R_3_ and R_4_. T_1_, R_5_, and R_6_ constitute the current source I. According to the previous analysis of the AC path, the current source does not get amplified as the AC signal, and its equivalent noise source is suppressed by the differential amplifier circuit as a common-mode signal, which can be ignored.

For the equivalent noise sources e_n1_ and e_n2_ of IF3602, the noise source is input from the gate, and the differential amplifier circuit can be equivalent to a common source amplifier circuit, and its amplification factor is $\left(-g_{m}R_{2}\right)/\left(1+g_{m}R_{4}\right)$. The sum of the output noise of e_n1_ and e_n2_ at the output of V_out1_ is as follows:

(3) $$\sqrt{2{\left(-g_{m}R_{1}\right)}^{2}e_{n1}{}^{2}/{\left(1+g_{m}R_{3}\right)}^{2}}$$

For thermal noise sources e_n3_ and e_n4_ of R_1_ and R_2_, the noise source PSD satisfies $\sqrt{4kTR_{1}}$ and $\sqrt{4kTR_{2}}$, *k* is the Boltzmann constant, and *T* is the temperature in Kelvin. These sources influence on the output end directly, and R_1_ and R_2_ are equal, so the sum of the output noise of e_n3_ and e_n4_ at the output end is as follows:

(4) $$\sqrt{2e_{n3}{}^{2}}$$

The PSDs of thermal noise sources e_n5_ and e_n6_ of R_3_ and R_4_ are $\sqrt{4kTR_{3}}$ and $\sqrt{4kTR_{4}}$, respectively, and their resistance values are equal. The thermal noise is input from the source of IF3602 and output at the drain after being amplified. In the circumstances, the differential circuit should be equivalent to a common-gate amplifying circuit, and its amplification factor is $g_{m}R_{1}/\left(1+g_{m}R_{3}\right)$. The sum of the output noises of e_n5_ and e_n6_ at the output is as follows:

(5) $$\sqrt{{2{\left(g_{m}R_{1}\right)}^{2}e_{n5}{}^{2}/\left(1+g_{m}R_{3}\right)}^{2}}$$

According to the circuit superposition theorem, the sum of the output noise of the IF3602 differential amplifier circuit at the output end is as follows:

(6) $$e_{\mathrm{nJFETout}}=\sqrt{2e_{n3}{}^{2}+2(e_{n1}{}^{2}+e_{n5}{}^{2}){\left(g_{m}R_{1}\right)}^{2}/{\left(1+g_{m}R_{3}\right)}^{2}}$$

Usually, to avoid the influence of circuit amplification on noise results, it is necessary to normalize the output noise by dividing it by the circuit’s amplification (i.e., the transfer function) and convert it into equivalent input noise to measure the noise floor of the circuit. The equivalent input noise of the differential amplifier circuit is as follows:

(7) $$e_{\mathrm{nJFETin}}=\frac{e_{\mathrm{nout}}}{\left|A_{v}\right|}=\sqrt{2e_{n1}{}^{2}+2e_{n5}{}^{2}+2e_{n3}{}^{2}{\left(1+g_{m}R_{3}\right)}^{2}/{\left(g_{m}R_{1}\right)}^{2}}$$

The equivalent noise source e_n1_ of the JFET is related to the static operating point, which can be regarded as a fixed value after referring to the minimum noise static operating point setting in the datasheet. The value of R_3_ is very small and leads to the thermal noise introduced into the input is extremely limited. Therefore, the optimization of resistor R_1_ should be focused. From Equation (6), the thermal noise of R_1_ resistance at the input terminal equivalently is as follows:

(8) $$e_{\mathrm{nR}1\mathrm{in}}=\sqrt{e_{n3}{}^{2}{\left(1+g_{m}R_{3}\right)}^{2}/{\left(g_{m}R_{1}\right)}^{2}}=\sqrt{4kT{\left(1+g_{m}R_{3}\right)}^{2}/g_{m}{}^{2}R_{1}}$$

When increasing the resistance value of R_1_, the input noise of R_1_ will decrease according to Equation (8), and the amplification factor will increase according to Equation (2). The whole optimization meets the requirements of the increasing the gain of the first stage and decreasing the noise floor of the circuit. However, increasing R_1_ will inevitably cause the offset of the static operating point of the circuit; therefore, the supply voltage of the amplifier circuit must be increased synchronously to roughly keep the static operating point of the JFET as described in the datasheet (V_DS_ = 3V, I_D_ = 5mA). After optimization, the parameters of the amplifier circuit shown in Figure 4 are finally determined as shown in Table 2.

### 3.5. Noise Testing and Analysis

The instrument can only measure the output noise power spectrum of the amplifier circuit. The actual noise floor of the sensing system needs to be converted through the transfer function. First, the transfer function of each level, the overall transfer function in Figure 4, needs to be calculated, and the results are shown in Equations (9)–(13) as follows:

(9) $$H_{circuit}(s)=\frac{V_{out}(s)}{V_{in}(s)}=\frac{V_{out}(s)}{V_{sig+}(s)-V_{sig-}(s)}=H_{1}(s)H_{2}(s)H_{3}(s)H_{4}(s)$$

(10) $$H_{1}(s)=A_{v}=\left(-g_{m}R_{1}\right)/(1+g_{m}R_{3})=\left(-g_{m}R_{2}\right)/\left(1+g_{m}R_{4}\right)$$

(11) $$H_{2}(s)=(-sC_{1}R_{11})/\left(1+sC_{1}R_{7}\right)=\left(-sC_{3}R_{12}\right)/\left(1+sC_{3}R_{9}\right)$$

(12) $$H_{3}(s)=R_{17}/{(R}_{13}{+R}_{15})=R_{18}/{(R}_{14}{+R}_{16})$$

(13) $$H_{4}(s)=1/\left(1+sC_{5}{(R}_{19}{+R}_{20})\right)$$

**Figure 10:**
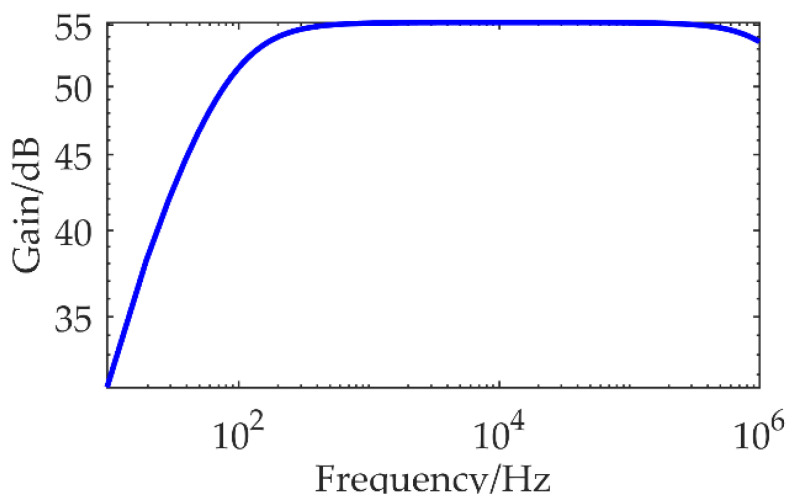
Overall amplitude-frequency characteristic curve of low-noise amplifier circuit.

The final transfer function can be calculated by substituting the parameters in Table 2 into Equation (9). The result is shown in Figure 10.

**Figure 11:**
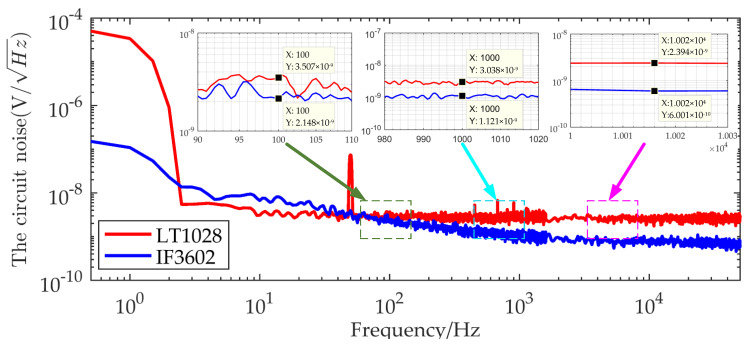
Comparison of the noise floor between the IF3602 circuit and the LT1028.

The gain of the IF3602 LNA circuit is kept stable at 55 dB in the 100 Hz–100 kHz. Because the PSD of the TEM low-frequency signal is large, if the gain at the full band is maintained stable, the signal will be more likely to saturate in the early stage. In this case, a non-ideal high-pass filter can be used to reduce the gain of the amplifier circuit for low-frequency signals, because the gain of the non-ideal high-pass filter is weak in the low frequency and large in the high frequency. A more accurate TEM secondary field signal can be recovered by adding a transfer function correction during data processing. The noise PSD at the output of the circuit was detected using an Agilent 35670A dynamic signal analyzer when shorted the input under EM shielding conditions. In order to record the noise floor of the amplifier circuit detail, the output noise floor of the IF3602 low-noise amplifier and LT1028 amplifier in the frequency bands of 0–800 Hz, 800–1600 Hz, and 1.6–52.8 kHz were collected and spliced. The equivalent input noise floor of the two is shown in Figure 11.

According to Figure 11, the noise floor of the IF3602 differential amplifier at 100 Hz, 1 kHz, and 10 kHz are $2.15\mathrm{nV}/\sqrt{\mathrm{Hz}}$, $1.12\mathrm{nV}/\sqrt{\mathrm{Hz}}$, and $0.60\mathrm{nV}/\sqrt{\mathrm{Hz}}$ respectively, which are lower than the corresponding frequency noise of the LT1028 as follows: $3.50\mathrm{nV}/\sqrt{\mathrm{Hz}}$, $3.04\mathrm{nV}/\sqrt{\mathrm{Hz}}$, and $2.39\mathrm{nV}/\sqrt{\mathrm{Hz}}$. The noise floor of the IF3602 circuit is significantly lower than the noise floor of the LT1028 circuit in the TEM band of 100 Hz–52.8 kHz.

**Figure 12:**
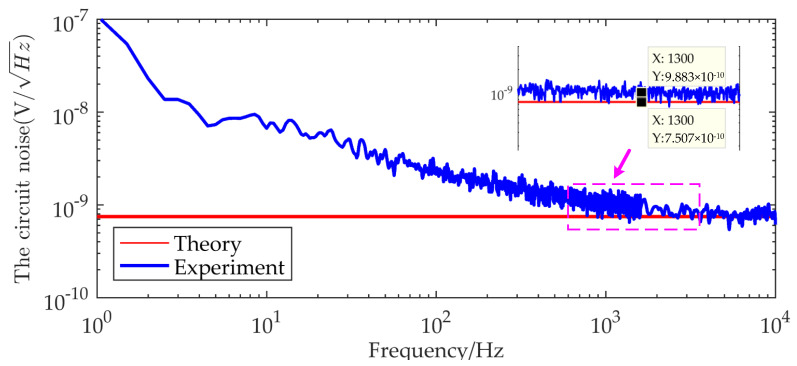
Comparison of measured noise data and theoretical calculated values of IF3602 low-noise amplifier circuit.

Figure 12 compares the actual noise curve of the IF3602 differential amplifier circuit with the calculated one based on Table 2 and Equation (7). The actual noise is close to the theoretical noise, and both of them reach below $1\mathrm{nV}/\sqrt{\mathrm{Hz}}$ above 1 kHz. However, the noise floor is different at low frequencies. The reason is that, when calculating the thermotical noise, only the corresponding noise of the device at 100 Hz given by the datasheet was used. However, the JFET is seriously disturbed by 1/*f* noise at low frequency in practice, and the amplitude of 1/*f* noise is inversely proportional to the frequency. So, in the high-frequency band, the theoretical noise is close to the actual noise, and the actual noise floor deviates from the theory.

## 4. Indoor Comparison Experiment

**Figure 13:**
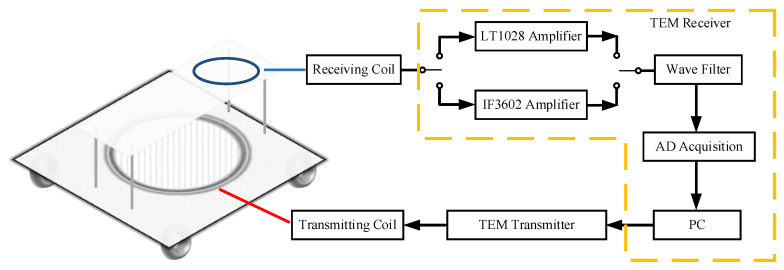
Schematic diagram of laboratory simulation comparison test.

**Figure 14:**
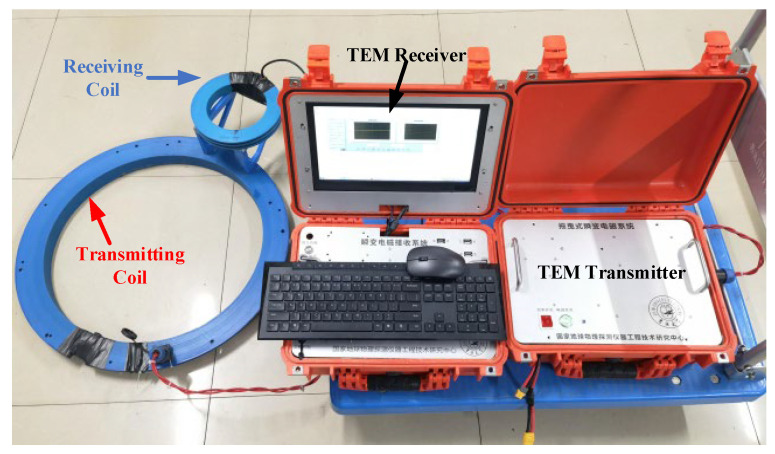
The laboratory simulation comparison test and TEM devices.

To further compare the performance of the IF3602 LNA in the receiver, we experimented with the simulation using a small-loop TEM device in the laboratory. Figure 13 shows the schematic diagram of the comparison test. The devices are shown in Figure 14, and the parameters of the device are as follows:

- The radius of the small loop transmitting coil used is 26.5 cm;
- The number of turns of the transmitting loop is 10;
- The transmitting current is 6 A;
- The radius of the receiving coil is 10 cm;
- The number of turns of the receiving loop is 32.

**Figure 15:**
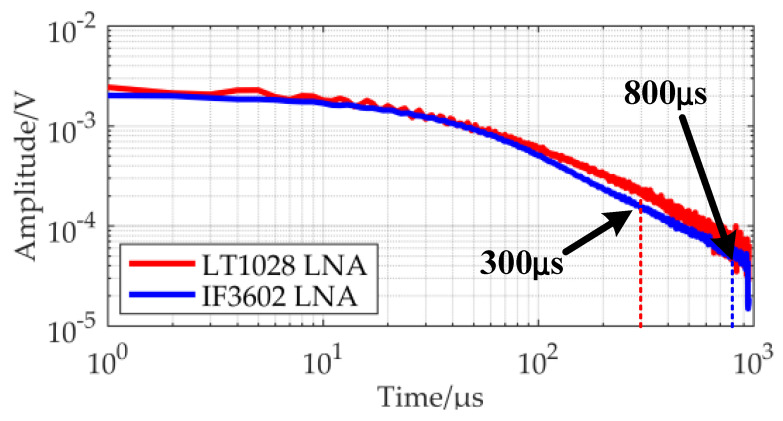
Comparison of the data observed by the receiving device in the laboratory.

To keep the circuit filter from interfering with data observation, a low-noise LT1028 amplifier board with an amplification factor of 10 was used as the pre-stage, and the same post-stage circuit as the IF3602 low-noise circuit was added, which was adjusted to have the same frequency band and similar gain as the IF3602 amplifier. Both groups of experiments were taken 128 times superposition average to avoid the influence of the amount of superposition on the quality of the received data. The results are shown in Figure 15.

According to the results, the IF3602 amplifier receiver has a smoother signal and higher data quality than the LT1028 amplifier receiver after taking a double logarithm. The effective data length of the IF3602 amplifier raw data is 800 μs, which is about 2.6 times longer than the LT1028 amplifier receiver.

The relationship between TEM detecting depth and detecting time is described using formula $h=\sqrt{2t/\sigma \mu_{0}}$, *σ* is the electrical conductivity of the geological body and *μ*0 is the vacuum magnetic permeability. Since the location of the experiment remains unchanged, the electrical conductivity values of the two geological bodies are the same, and the vacuum magnetic permeability is a fixed value, so the performance of the system is only determined by the effective length of the data. The receiver with the IF3602 amplifier increases the TEM theoretical maximum detection depth to about 1.6 times that of the original system, according to the result and the TEM detecting depth formula. In addition, because of the smoother performance of the IF3602 receiver observed data under 128 times of superposition, it is suggested that the number of superpositions can be reduced to have the same SNR as the LT1028 amplifier receiver while still accessing the higher detection efficiency.

## 5. Discussion

The IF3602 LNA has better noise characteristics than the current TEM common LT1028 LNA, and its noise reduces to $0.60\mathrm{nV}/\sqrt{\mathrm{Hz}}$ at 10 kHz. By comparing the IF3602 LNA TEM receiver and the LT1028 LNA TEM receiver, the observed signal of the receiver with the IF3602 LNA has a significant improvement in effective data length and data quality. The detection depth is increased to 1.6 times that of the former LT1028 system. However, we hold that there are still the following problems, which limit its application and use effect.

Due to the limitations of the JFET device manufacture, the parameters between the devices are quite different, which requires strict pairing before use. Even if the IF3602 used in the experiment has been paired, there are still differences in the internal JFET. The symmetry of the circuit is not as good as that of the ideal differential circuit. The ignored current source also introduces circuit noise. Although the IF3602 has a pair of paired JFETs built-in, its high cost and its inside JFETs parameter difference limit the application of low-noise JFETs.

However, we hold that the above problems can be ameliorated by the following improvements. Before the circuit is manufactured, it is recommended to use a graphic instrument to test the JFET parameter curve and try to select a JFET with similar parameters. If the test conditions are not available, i.e., the parameter characteristic curve cannot be tested, first, a simple circuit can be designed about the minimum noise static operating point in the datasheet, then measure the actual static operating point of each JFET, and select a JFET with good consistency. This can effectively reduce the introduction of common-mode circuit noise caused by JFET parameter difference. Matching can effectively reduce the demand for JFETs such as the IF3602 that have been matched by manufacturers and can effectively reduce the cost of JFET LNA circuits.

## 6. Conclusions

In order to improve the survey capability and accuracy of the TEM system, we have designed a low-noise differential amplifier for the TEM receiver by using the low-noise JFET IF3602 according to the power spectrum characteristics of the TEM forward response. Additionally, effectively increase the effective length and quality of the TEM observed data.

Firstly, based on the model of TEM forward response, we created the theoretical signal by homogeneous half-space. Secondly, we analyzed the power spectrum characteristics of the TEM signal to determine the requirements of the TEM amplifier noise floor and frequency bands. Thirdly, we designed a source-coupled differential amplifier circuit using the IF3602 low-noise JFET. We applied a current source to replace the resistance in the conventional long-tail circuit for providing stable current output for the left and right branches to simplify the JFET static working point settings. Besides, the parameters of the low-noise circuit were optimized combined with the theoretical model of the circuit and noise. Results of the noise test indicate that the designed circuit achieves good noise characteristics performance with a minimum of $0.60\mathrm{nV}/\sqrt{\mathrm{Hz}}\cdots @10\mathrm{kHz}$, which is significantly lower than the performance of the corresponding frequency point of the LT1028 LNA. It can improve the ability of the system to distinguish TEM signals. Compared with the LT1028 LNA, the designed IF3602 low-noise circuit can increase the effective data points of the observed data by about 2.6 times and the theoretical detection depth by about 1.6 times under the same experimental conditions. In addition, under the condition of the same number of superpositions, the signal amplified by the IF3602 LNA is of high quality and smoother, which proves that reducing the noise floor of the preamplifier can not only improve the data quality but also greatly help improve the survey capability and detection efficiency.

Therefore, this study is of great significance for the design of preamplifier circuits in TEM receivers. In future work, we will continue to explore new circuit design schemes, such as parallel noise reduction design, to further improve the detection performance of the TEM receivers.

## Figures and Tables

**Table 1:** Comparison of noise performance of low-noise devices.

Device Model	$e_{n}\;(nV/\sqrt{Hz})$	$i_{n}\;(pA/\sqrt{Hz})$
AD745	3.2 (*f* = 1 kHz)	0.007 (*f* = 1 kHz)
AD797	0.9 (*f* = 1 kHz)	2.0 (*f* = 1 kHz)
AD8672	2.8 (*f* = 1 kHz)	0.3 (*f* = 1 kHz)
OPA847	0.92 (*f* > 1 MHz)	3.5 (*f* > 1 MHz)
INA163	1.0 (*f* = 1 kHz)	0.8 (*f* = 1 kHz)
LT1028	0.85 (*f* = 1 kHz)	1.0 (*f* = 1 kHz)
LSK389	0.9 (*f* = 1 kHz)	——
IF3602	0.5 (*f* = 100 Hz)	——

**Table 2:** Circuit device parameters.

Components	Component Type	Component Value
R_1_, R_2_	1% Resistor 2.4 kΩ 0805 × 2	1.2 kΩ
J_1_, J_2_	IF3602 Low Noise N-Channel JFET Pair	InterFET
R_3_, R_4_	1% Resistor 0805	1 Ω
T_1_	BJT	2N1711
R_5_	1% Resistor 0805	0 Ω
R_6_	1% Resistor 3.6 kΩ 0805 × 4	900 Ω
C_1_, C_3_	4.7 μF Y5V 0805 Capacitor × 4	18.8 μF
C_2_, C_4_	Y5V 0805 Capacitor	10 nF
R_7_, R_9_	1% Resistor 0805	510 Ω
R_8_, R_10_	1% Resistor 0805	100 Ω
A_1_, A_2_	Low Noise IOA	LT1028
R_11_, R_12_	1% Resistor 0805	5.1 kΩ
R_13_, R_14_, R_19_	1% Resistor 100 Ω 0805 × 2	50 Ω
R_15_, R_16_, R_17_, R_18_	INA105 Built-in Resistor	25 kΩ
A_3_	Precision Difference Amplifier	INA105
R_20_	1% Resistor 0805	910 Ω
C_5_	Y5V 0805 Capacitor	860 pF
V_DD_	Lithium Battery	+12 V
V_SS_	Lithium Battery	−12 V

## Data Availability

Upon reasonable request, the data supporting this investigation are available from the corresponding authors.
